# Posttransplant Lymphoproliferative Disorder in a Patient With Alpha-1 Antitrypsin Deficiency: A Case Report

**DOI:** 10.7759/cureus.62107

**Published:** 2024-06-10

**Authors:** Lauren Joly, Richard Virgilio, Claire Yother

**Affiliations:** 1 Pediatrics, Edward Via College of Osteopathic Medicine, Auburn, USA; 2 Clinical Affairs, Edward Via College of Osteopathic Medicine, Auburn, USA; 3 Pediatric Medicine, Gadsden Pediatric Clinic, Gadsden, USA

**Keywords:** posttransplant lymphoproliferative disorder, ebv-associated lymphoma, infectious mononucleosis-like nondestructive ptld, nondestructive ptld, solid organ transplant, ebv ptld, alpha-1-antitrypsin deficiency

## Abstract

A 13-year-old male with a past medical history of receiving a whole liver transplant secondary to alpha-1 antitrypsin deficiency (AATD) with subsequent inferior vena cava thrombosis nine years prior presented to the emergency department with abdominal distension, shortness of breath, coughing, and left superficial cervical lymphadenopathy. He had seen his pediatrician the day before where he tested negative for group A *Streptococcus*, influenza, and severe acute respiratory syndrome coronavirus 2. Additionally, the patient reported having elevated liver function tests noted from the results of lab tests taken earlier that day. The patient was admitted to the hospital. While at the hospital, a lymph node biopsy was performed, and pathology from that biopsy revealed infectious mononucleosis-like nondestructive posttransplant lymphoproliferative disorder (PTLD). Due to the patient's liver transplant nine years prior, the patient was on an immunosuppressant medication: tacrolimus 2 mg. To treat the PTLD, the tacrolimus was reduced, then stopped, and then subsequently restarted at 1 mg. He also was given ganciclovir and prednisone. Two months after recovering from the PTLD, the patient’s Epstein-Barr-virus (EBV) viral load continued to fluctuate, and he was treated with three doses of the monoclonal antibody drug rituximab. After treatment with rituximab, his EBV viral load remained stable. This case report gives insight into the treatment of PTLD and can serve as a reminder to be aware of the possibility of PTLD in a pediatric patient with AATD multiple years after a transplant.

## Introduction

Posttransplant lymphoproliferative disorders (PTLDs) are a collection of life-threatening complications that involve uncontrolled proliferation of lymphoid cells as a consequence of extrinsic immunosuppression after organ or hematopoietic stem cell transplantation [1]. PTLD has most commonly been associated with Epstein-Barr virus (EBV) [1,2]. For transplant recipients who use immunosuppressive drugs, their EBV-specific cytotoxic t-cell response is suppressed [3]. This puts these patients at a higher risk of developing EBV. The incidence of PTLD in patients with a previous transplant is estimated to be 1-33% [3]. There are two peaks of PTLD incidence that have been noted [4]. The first, more common peak is in the first posttransplant year (mostly EBV seropositive), and the second peak is between the fifth and 15th year after transplant (mostly EBV seronegative) [4]. Interestingly, primary EBV infection is the most common cause for PTLD development in pediatric patients [4]. There appears to be an increased risk of PTLD seen in young recipients, presumably due to the high percentage who are EBV naive at transplantation, leaving them susceptible to primary infection [5]. However, for adults, the first year after transplantation is when the incidence is highest [6].

This case will discuss the complications of PTLD in a pediatric patient with alpha-1 antitrypsin deficiency (AATD) who received a liver transplant nine years prior to the onset of PTLD. AATD is an autosomal recessive disorder marked by very low levels of alpha-1 antitrypsin [7]. It is one of the most common genetic disorders of the Caucasian population [8]. Patients usually present in early infancy with liver disease or emphysema in adulthood [8]. The explanation for the liver disease relates to the fact that hepatocytes are the main location of alpha-1 antitrypsin production and that mutations of the alpha-1 antitrypsin gene cause errors in the processing of alpha-1 antitrypsin, resulting in hepatocyte injury [9,10].

The most common symptoms of PTLDs include fever, chills, weight loss, fatigue, and lymphadenopathy [3]. Some PTLDs present clinically with just nonspecific symptoms, while other types of PTLDs present with symptoms such as lymphadenopathy and hepatosplenomegaly [3]. As PTLD progresses, it can spread to any organ, including bone marrow, liver, spleen, kidney, and even the central nervous system in rare cases [3]. Histological examination of tumor tissue is the gold standard for PTLD diagnosis. Furthermore, an excisional biopsy or resection of a whole lymph node is preferred over a core needle biopsy [3]. According to the World Health Organization (WHO) 2017 classification, PTLD can be categorized as either nondestructive PTLDs (plasmacytic hyperplasia, florid follicular hyperplasia, infectious mononucleosis-like PTLD), polymorphic PTLD, monomorphic PTLD (B-cell, T-cell, natural killer cell types), or classic Hodgkin's lymphoma-like PTLD [4]. The Children’s Hospital of Pittsburgh recommends that the first step to treat a pediatric transplant recipient with EBV-related PTLD should be to stop immunosuppressant drugs, initiate ganciclovir or CytoGam® (Kamada Therapeutics), and monitor liver function tests and quantitative EBV polymerase chain reaction (PCR) results [11]. In addition, rituximab can be used to treat PTLD [3]. It is important to note that the effectiveness of rituximab is usually lost if it is used for an extended period of time [3]. Consequently, it is recommended that rituximab use should be limited to once weekly for up to four doses [3]. Once the patient recovers, there is no clear consensus on what preventative measures should be taken [11]. According to the Children's Hospital of Pittsburgh, it is common for patients to note rebound increases in their EBV viral load after recovery from PTLD [11]. A portion of the rebounds appeared to correlate with the reintroduction of immunosuppressant drugs [11]. These rebounds have rarely been associated with evidence of recurrence of EBV-related PTLD in patients treated at the Children’s Hospital of Pittsburgh [11]. Since the frequency of rebound is remarkably high and the rate of actual recurrent PTLD is considerably low, it is not required to routinely monitor the EBV viral load after recovering from PTLD [11]. However, other studies have found monitoring the viral load beneficial. Data from the pediatric field of liver transplantation have found that monitoring EBV viremia can allow practitioners to reduce immunosuppressants and prevent diagnosis of PTLD [12]. Again, there is no clear consensus on a correct management plan to prevent the redevelopment of PTLD [11], so treatment should be carefully decided on a case-by-case basis.

## Case presentation

A 13-year-old male presented to the emergency department of a pediatric hospital with a chief complaint of fever, swollen lymph nodes, and elevated liver function tests. Additionally, he complained of dyspnea, cough, back pain, and abdominal swelling. The patient had a past medical history of having a whole liver transplant with the Roux-en-Y technique at the age of four, secondary to AATD with subsequent inferior vena cava thrombosis. The patient's mother reported that the patient’s fever started one day ago and reached a maximum of 101.7°F. He was seen at his pediatrician’s office the day before, where he tested negative for severe acute respiratory syndrome coronavirus 2, influenza, and group A *Streptococcus*. The patient’s mother also reported that his liver transplant team’s nurse practitioner ordered lab tests to be drawn. These tests revealed elevated liver function tests. The patient denied any trauma or injury to his back, vomiting, diarrhea, dysuria, or sinus congestion. He reported that he was diagnosed with the flu two weeks ago and noted swollen lymph nodes on the left side of the neck at the beginning of the viral illness and an increase in swelling over the last two weeks. The patient’s current medications included cetirizine 10 mg oral tablet taken once daily, ondansetron 4 mg oral tablet taken as needed for nausea or vomiting, tacrolimus 2 mg taken once a day, and rizatriptan 10 mg taken as needed at headache onset.

On the physical exam, the patient was well-appearing, nontoxic, interactive, and in no distress. Left superficial posterior cervical lymphadenopathy with no tenderness to palpation was noted. His lungs were clear to auscultation bilaterally. Tachypnea, tachycardia, bilateral costovertebral angle tenderness, right upper quadrant tenderness to palpation, and abdominal distention were noted on physical exam. No organomegaly was noted on the physical exam. The findings were concerning for PTLD due to the lymphadenopathy, constitutional symptoms, and history of transplantation. The findings were also concerning for solid organ transplant rejection and viral illness. Due to these differential diagnoses, the patient was considered high risk and was admitted to the hospital.

On the first day of admission, the patient was treated empirically with antibiotics, (piperacillin and tazobactam), along with intravenous fluids for acute kidney injury. A hematologist/oncologist, transplant surgeon, and otorhinolaryngologist (for posterior cervical lymphadenopathy) were consulted. The following morning, piperacillin and tazobactam were discontinued and transitioned to ciprofloxacin and metronidazole due to the nephrotoxicity of piperacillin and tazobactam. A computed tomography (CT) of the chest, abdomen, and pelvis showed bilateral pleural effusions, trace pericardial effusion, splenomegaly, and pelvic predominant ascites. By the third morning, the patient's EBV viral load was elevated at 17,580 international units; therefore, he was started on ganciclovir. On the fourth day, the patient’s tacrolimus levels were found to be elevated. Due to this, the tacrolimus dose was reduced from 2 mg to 1 mg. Additionally, a CT head with contrast was performed to evaluate and plan for posterior cervical lymph node excisional biopsy.

By the fifth day, the patient received one unit of platelets. Next, interventional radiology completed a liver biopsy and a thoracentesis, removing 1 liter of fluid. While still in the same operating room, a left posterior cervical lymph node was surgically excised and biopsied by the otorhinolaryngologist. After the procedures, the patient received furosemide and albumin. Tacrolimus was held due to continued elevated tacrolimus levels. By the end of the sixth day, the patient was able to restart 1 mg tacrolimus, and the tacrolimus levels were monitored throughout the rest of his admission.

On the eighth day, the patient was started on oral clindamycin for phlebitis of the right antecubital IV site where IV ganciclovir was infused. He was transitioned to oral ganciclovir. On the ninth day, a positron emission tomography scan was obtained, revealing no lymphadenopathy. Also, pathology from the lymph node biopsy was consistent with infectious mononucleosis-like nondestructive PTLD. His liver biopsy showed portal and sinusoidal inflammation with features of endotheliitis and bile duct damage. The findings from the liver biopsy were ruled to either be due to acute rejection or due to primary EBV infection. The patient was continued on oral clindamycin and oral ganciclovir. He was started on prednisone 40 mg daily. On the eleventh day, he was deemed medically stable for discharge. He was discharged from the hospital to his home with instructions to continue clindamycin, take ganciclovir for an additional seven days, and continue to take and taper prednisone. He was also given instructions to follow up with the hematologist/oncologist and his transplant team as an outpatient.

The patient continued to have frequent lab and clinic visits while on the oral steroid taper. His EBV viremia continued to fluctuate. His hematologist/oncologist treated him with rituximab since his EBV viremia continued to vary. He received rituximab once weekly for a total of three doses, and his EBV viral load has remained stable. 

The patient was seen by his pediatrician approximately 17 months after his original presentation with PTLD. The patient reports that he is still taking 1 mg of tacrolimus and his transplant team is continuing to monitor his liver function tests and quantitative EBV PCR results every one to two months. A summarized timeline of the patient's treatment regimen can be seen in the figure. 

**Figure 1:**
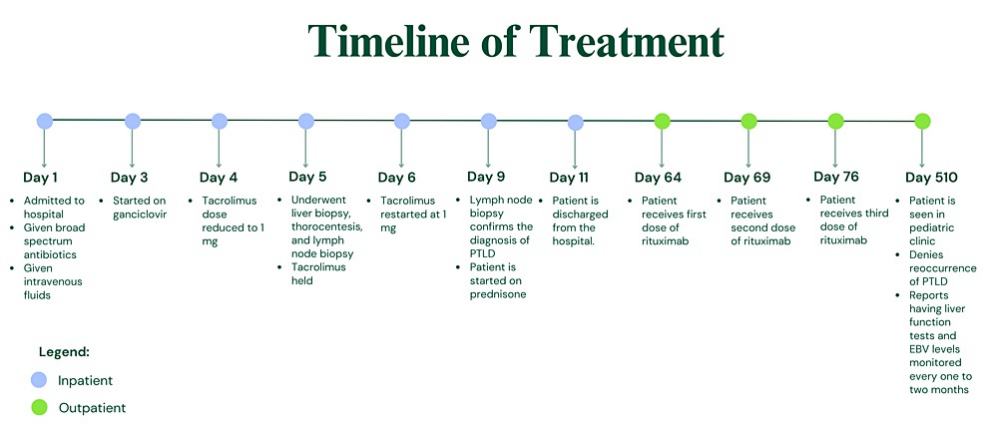
Summarized timeline of the treatment regimen PTLD: Posttransplant lymphoproliferative disorder; EBV: Epstein-Barr virus

## Discussion

In this case, PTLD occurred in a patient with AATD, nine years posttransplant. AATD is one of the most common genetic disorders in the Caucasian population [8], yet there appears to be sparse scholarly articles on the association of PTLD and AATD. This case is likely not the only co-occurrence of PTLD and AATD, due to the prevalence of transplantation in AATD and the rise of PTLD [4]. This case provides an example for likely future similar cases.

As stated earlier, there are two peaks of incidence of PTLD that occur. One is in the first year after transplantation, and one is five to 15 years after transplantation [4]. It is worth noting that this patient did not have the development of PTLD in his first year of transplantation, which is the more common of the two peaks [6]. Instead, this patient developed PTLD in that second peak. Developing PTLD in the second peak was associated with patients who were EBV naive prior to transplantation and subsequently developed a primary EBV infection after transplantation and immunosuppression [4]. Furthermore, the most common cause of PTLD in pediatric patients is primary EBV infections [4]. Therefore, it is likely that this patient was EBV seronegative up until the ninth posttransplant year when he developed the PTLD.

As mentioned earlier, PTLDs are classified into different types: nondestructive, polymorphic, monomorphic, and Hodgkin lymphoma-like [4]. In this case, pathology revealed a nondestructive PTLD. This type of PTLD is a more favorable type, and the patient was eventually managed as recommended by stopping his immunosuppressants, starting him on the ganciclovir, and monitoring liver function tests and EBV viral load [11]. However, it is important to note that there was a delay in this care. Due to his health care team being concerned for organ rejection, the immunosuppressant was not reduced and then subsequently stopped until the fourth and fifth day, respectively. This is a common conundrum that practitioners face when treating patients with PTLD: is this acute organ rejection or PTLD? What makes this question even more challenging is the dangerous outcome that could happen with treatment. Stopping immunosuppressant medication carries the risk of transplant rejection. So, practitioners need to be able to rule out acute rejection before treating PTLD. They also must be aware of the risk of developing acute cellular rejection after stopping immunosuppressants. For example, it has been found that adults with PTLD have mortality rates up to 60% [11]. A majority of those deaths were caused by chronic rejection resulting from the reduction of immunosuppressants in the effort to treat PTLD [11]. However, children seem to have a much more successful recovery period from PTLD [11]. The long-term survival rates for pediatric patients with PTLD is between 60% and 100% [11]. They specifically noted death due to rejection being infrequent in these pediatric cases [11]. Despite this hopeful statistic, for many cases of PTLD, deciding on whether to reduce or withdrawal immunosuppressants still proves to be quite complicated. For most cases, treatment must be considered on an individual basis. For example, in one retrospective analysis of 335 pediatric patients with a history of a liver transplant, it was found that immunosuppressant medication could be safely stopped in 19 cases of PTLD. They reported that acute rejection was an acceptable and manageable risk for those individual cases and reported no deaths as a result of this immunosuppression [12]. Similarly for this case, acute rejection was considered a risk but ruled an acceptable and manageable one. Furthermore, as mentioned earlier, the liver biopsy in this case did not rule out acute rejection. The portal and sinusoidal inflammation with features of endotheliitis and bile duct damage was actually ruled to be either due to acute rejection or due to primary EBV infection. While it is common for acute organ rejection to be on practitioner’s differential diagnosis list for PTLD cases, it is not likely that the two will present at the same time due to the contradiction of the two’s existence together: if the patient is on enough immunosuppressant, they will not have organ rejection but could develop PTLD, and if the patient is not on enough immunosuppressants, this will decrease their chance of developing PTLD but increase their chance of acute rejection. So, this raises the next question: did the patient in this case have acute rejection due to the halting of his immunosuppressant? Since the patient’s liver function levels were already elevated prior to admission and stopping the immunosuppressants, it is more likely that the findings from the biopsy were indicative of primary EBV infection, instead. With all that said, the decision to reduce or stop immunosuppressants should be carefully decided on an individual basis.

After reducing or stopping immunosuppressant medication, the next step in treating PTLD is adding the antiviral drug, ganciclovir. The patient in this case was not started on ganciclovir until the third day of admission. As for the rituximab, not all patients being treated for PTLD require it [11], but the patient in this case was given rituximab due to rising EBV viral load. In a study of pediatric patients with PTLD, rituximab was found to have a response rate of 65% [11]. The patient’s response in this case report aligns with the majority, as he also had a successful response rate to rituximab.

Finally, the last form of treatment for PTLD that the patient received, and continues to receive, is outpatient serial lab tests to monitor his EBV viral load. As mentioned earlier, it is common for EBV PCR results to increase after the resolution of PTLD, but this does not necessarily mean that the need to continue to monitor the EBV viral load is present [11]. The Children’s Hospital of Philadelphia actually recommends against it [11]. On the other hand, other sources have spoken of the benefits of monitoring the viral load, like being able to use the quantitative EBV PCR results to help manage lowering the immunosuppressant dose to prevent the development, or redevelopment, of PTLD [12]. There are no clear guidelines on how to prevent the redevelopment of PTLD [11], and physicians must, once again, use their best judgment and make these decisions on an individual, case-by-case basis.

Clearly, the patient in this report had a good response to treatment. This patient’s outcome was similar to patients who also had a nondestructive form of PTLD. This form of PTLD has a great prognosis compared to others [13]. In one more specific study that observed the management of PTLD in pediatric liver transplant recipients who were taking tacrolimus, the long-term survival rate was 78% [13]. Of the patient deaths, most of them were from PTLD itself, and it was emphasized that these PTLD deaths were all advanced cases with multiple sites of PTLD at the time of diagnosis [13]. 

## Conclusions

In this case study, we discussed the diagnosis of PTLD through lymph node biopsy, the treatment of PTLD with the cessation and subsequent lower dosage of immunosuppressants, the use of ganciclovir, prednisone, rituximab, and the follow-up of the patient with serial EBV viral load monitoring. We were able to make note of the possible complications that come with treating PTLD and how this patient’s outcome compared to other pediatric patients in similar circumstances. With this information, we hope that clinicians can be more prepared for the likely occurrence of more pediatric posttransplant patients who present to their doctors with similar symptoms and that clinicians will be more readily equipped to treat them.

## References

[1] Dharnidharka VR, Webster AC, Martinez OM, Preiksaitis JK, Leblond V, Choquet S (2016). Post-transplant lymphoproliferative disorders. Nat Rev Dis Primers.

[2] Nijland ML, Kersten MJ, Pals ST, Bemelman FJ, Ten Berge IJ (2016). Epstein-Barr virus-positive posttransplant lymphoproliferative disease after solid organ transplantation: pathogenesis, clinical manifestations, diagnosis, and management. Transplant Direct.

[3] Fujimoto A, Suzuki R (2020). Epstein-Barr virus-associated post-transplant lymphoproliferative disorders after hematopoietic stem cell transplantation: pathogenesis, risk factors and clinical outcomes. Cancers (Basel).

[4] Abbas F, El Kossi M, Shaheen IS, Sharma A, Halawa A (2020). Post-transplantation lymphoproliferative disorders: current concepts and future therapeutic approaches. World J Transplant.

[5] Quinlan SC, Pfeiffer RM, Morton LM, Engels EA (2011). Risk factors for early-onset and late-onset post-transplant lymphoproliferative disorder in kidney recipients in the United States. Am J Hematol.

[6] Gandhi S, Behling E, Behrens D, Ferber A, Schwarting R, Budak-Alpdogan T (2020). Late-onset posttransplant lymphoproliferative disorders after solid organ transplantation in adults: a case series and review of the literature. Case Rep Transplant.

[7] Robbins SL, Cotran RS, Kumar V, Abbas AK, Aster JC, Fausto N (2010). Chapter 18 liver and biliary tract. Pathologic Basis of Disease (8th Edition).

[8] Prachalias AA, Kalife M, Francavilla R (2008). Liver transplantation for alpha-1-antitrypsin deficiency in children. Transpl Int.

[9] Crystal RG (1990). Alpha 1-antitrypsin deficiency, emphysema, and liver disease. Genetic basis and strategies for therapy. J Clin Invest.

[10] Teckman JH, Blomenkamp KS (2017). Pathophysiology of alpha-1 antitrypsin deficiency liver disease. Methods Mol Biol.

[11] Green M (2001). Management of Epstein-Barr virus-induced post-transplant lymphoproliferative disease in recipients of solid organ transplantation. Am J Transplant.

[12] Dufour JF, Fey MF (2006). What is the current treatment of PTLD after liver transplantation?. J Hepatol.

[13] Cacciarelli TV, Green M, Jaffe R, Mazariegos GV, Jain A, Fung JJ, Reyes J (1998). Management of posttransplant lymphoproliferative disease in pediatric liver transplant recipients receiving primary tacrolimus (FK506) therapy. Transplantation.

